# Excerpts from Electrophysiology Sessions at the European Society of Cardiology Congress 2002 - Berlin

**Published:** 2003-01-01

**Authors:** Ashish Nabar

**Keywords:** European, Cardiology, Electrophysiology

## Current clinical problems encountered with implantable cardioverter defibrillators (ICDs)

The current scope of dual chamber ICD was discussed. It seems that 50% of the implants in US and 30% in Europe consist of a dual chamber device. Factors which influence the selection of a dual-chamber device include: 1) presence of an anti-bradycardia pacing indication, 2) need to discriminate supraventricular (SVT) from ventricular tachycardia (VT), 3) prevention and therapy of SVT and 4) presence of heart failure. Algorithms from various manufacturers discriminate SVT from VT with a very high sensitivity, but the specificity varies from 70-100%, thus not a dramatic improvement over the single-chamber ICDs. A German study that compared single- versus dual-chamber ICD did not show a clear benefit with respect to inappropriate therapy. In conclusion, dual-chamber ICDs are definitely useful when class I pacemaker indication is concomitantly present, are possibly useful in the presence of slow VTs, intermittent SVTs, presence of heart failure NYHA III-IV and are definitely not indicated in the presence of chronic atrial fibrillation (AF).

ICD functions have now expanded to: 1) good monitoring, 2) bradycardia support, 3) VT prevention and therapy, 4) ventricular fibrillation termination, 5) AF algorithms and 6) resynchronization. There was a plea that electrophysiologists should do device programming by themselves and not leave it to the company representative. It is true that device programming consumes a lot of office-time (20-25 minutes) and therefore devices should be simplified. This is also in the interest of cost reduction.

The incidence of ICD lead dislocation (atrial) or fracture (in the costo-clavicular region) is around 4.5%. Coaxial leads are at increased risk. There was no difference in the incidence of lead dysfunction with respect to single- or dual-chamber device. Pacing parameters can easily detect lead malfunction and DFT testing is usually not indicated. Inappropriate shock as a result of lead malfunction occurs in about 40% of cases, while failure to defibrillate in 3%. Other problems include oversensing, undersensing and increase in pacing threshold. Indications and techniques of extraction of a malfunctioning lead are not widely established and information is derived from small studies. The longer the lead is in-situ the larger the chance that excimer laser will be required for extraction. Open-chest surgery is rarely used, except in cases where a lot of thrombus is present all over the lead.

Understanding the effects of concomitant anti-arrhythmic drug (AAD) therapy in patients with ICD is important. AADs are frequently used when the ICD is implanted for VT and less frequently used when cardiac arrest or syncope is the indication. In the AVID study, 18% of the patients in the ICD-arm required AADs, in 2/3rds of the cases because of frequent shocks. Amiodarne was used in 40% of the instances. Pacifico et al., showed that Sotalol reduced the risk of death or delivery of the first shock by 48%. In general, class I agents increase the DFT, while class III agents except amiodarone decrease the DFT. The common reasons to continue AAD therapy after an ICD implant include: 1) decrease the frequency of VT/ventricular fibrillation, 2) decrease non-sustained VTs, 3) lower the VT rate so as to increase the effectiveness of ATP therapy, 4) suppress SVT, 5) control ventricular response to AF and 6) blunt exertional sinus tachycardia. An inappropriate diagnosis of VT during AF with fast ventricular rate can be unmasked by using beta-blockers. These could slow the ventricular rate, thus revealing the irregularity of the ventricular response. Further, AADs may increase the pacing threshold or slow the VT thereby preventing detection. Therefore, radiofrequency ablation should be considered in patients with recurrent slow VTs after ICD implantation. In routine practice, most centers do not evaluate the DFT after addition of AADs because of sufficient margin of safety provided by the maximal programmable shock.

## Biventricular pacing - a resynchronization therapy

Evidence suggests that biventricular pacing (BVP) could be classified as a class IA indication for patients with advanced heart failure in sinus rhythm, having a QRS width > 130 ms and who are optimally treated. BVP is definitely useful in patients belonging to NYHA class III-IV, but is not yet indicated for patients in functional class II. Above an intrinsic QRS width > 130 ms or paced QRS width > 200 ms there is no incremental benefit of BVP with worsening of QRS width. Patients with systolic LV dysfunction (EF < 35% and LVEDD > 55 mm), of either idiopathic or ischemic nature, but not diastolic dysfunction are candidates for this therapy. An aortic pre-ejection delay > 140 ms and an intra-ventricular conduction delay > 40 ms are agreed upon as the echocardiographic selection criteria. Role of BVP in patients with RBBB, left hemifascicular block or severe right ventricular dysfunction is not clear. Optimal therapy includes ACE inhibitors, beta blockers, digoxin, loop diuretics and spironolactone. Improvement after initiation of BVP is immediate and an "on-and-off" effect is demonstrable. Mechanical remodeling has not yet been documented. Implantation success rates close to 100% have been reported. LV lead placement is difficult in 20% of cases and coronary sinus perforation has been reported in 1-8%.

The MUSTIC 2 year follow-up data showed sustained clinical efficacy of biventricular pacing in patients with advanced heart failure (HF), with left ventricular (LV) systolic dysfunction and interventricular conduction disturbances, who were in sinus rhythm. No change in medical therapy, except for diuretics, was allowed during the study period. The mean NYHA class (2.9 to 2.1), 6-minute walk test (330 meters to 386 meters) and quality of life scores (48 to 29) showed significant improvement. The LV dimensions decreased (systolic by 6% and diastolic by 10%), but the 2-year follow-up results of LV ejection fraction (radionuclide) and peak VO2 are awaited. The biventricular paced QRS width was reduced as compared to the initial width, but the intrinsic QRS width remained unaltered. The 2-year survival was 76%. Similarly, the 6-month follow-up data from the MIRACLE trial showed favorable trends or a statistically significant reduction in the risk of worsening heart failure, total days hospitalized, duration of hospital stay per admission and need of intravenous medication. Effect on combined morbidity and all-cause mortality needs to be analyzed in larger prospective trials. In another presentation, cardiac resynchronizaton therapy was calculated to be a cost-effective treatment of heart failure. Yet it must be remembered that 20% of the patients are classified as non-responders of this therapy.

It was proposed that the benefits of treatment should be assessed according to the NYHA class prior to commencing biventricular pacing. Hemodynamic improvement and decrease in hospitalization should be evaluated in NYHA class IV patients. In addition, quality of life, functional capacity and survival needs assessment in patients in NYHA class III. In patients belonging to NYHA class II, it is the evaluation of disease progression and survival that is important.

## Post-operative Tetralogy of Fallot - is the electrophysiologist listening?

Less than 10% of patients with Tetralogy of Fallot (TOF) would survive longer than 10 years without surgery. Recent data indicates that the first 25 years following total correction is a low hazard phase, after which is the late hazard phase. Sudden cardiac death following TOF repair in a truly asymptomatic patient is rare. Avoiding ventriculotomy is not always possible. In an autopsy series of post-operative TOF, 6/48 deaths were reported to be sudden. Four of these 6 patients died after exertion. AV conduction system was normal and fibrosis was observed in the RBB. Myocardial fibrosis and islands of viable tissue were observed in the ventricular wall, a possible basis for reentrant arrhythmias. AF is observed in 15% of the cases and VT in 17% of the patients. A VT with LBBB morphology occurs late after repair of TOF. Bifascicular block is not associated with increased mortality.

Older age at surgery and high right ventricle (RV) to LV ratio have an impact on post-surgical mortality. Trans-annular patch and a non-restrictive RV physiology is the worst combination. Annual QRS width can help to monitor RV dilataion. Pulmonary regurgitation is the most important determinant of post-operative mortality. An implantable valved stent is an upcoming development in this regard. Application of cardiac resynchronization therapy, as in patients with LV systolic dysfunction and LBBB morphology, seems to be difficult considering the caterpillar-like sequential nature of RV contraction. A decrease in LV function could occur as a result of LV fibrosis.

